# Machine learning identification of biomarkers for vascular endothelial damage in obstructive sleep apnea

**DOI:** 10.1016/j.clinsp.2026.101165

**Published:** 2026-09-22

**Authors:** Tiantian Su, Song Shi

**Affiliations:** ENT Department, Tongren Hospital, Shanghai Jiao Tong University School of Medicine, Shanghai, China

**Keywords:** Obstructive sleep apnea, Vascular endothelial damage, Feature gene, Machine learning

## Abstract

**Background:**

Obstructive Sleep Apnea (OSA) is a common sleep disorder that, if left untreated, may lead to chronic damage to multiple organs. The purpose of this study is to use machine learning methods to identify biomarkers associated with Vascular Endothelial Damage (VED), providing insights for early diagnosis and targeted therapy of OSA.

**Methods:**

The authors conducted a differential expression analysis on the dataset GSE75097 to obtain Differentially Expressed Genes (DEGs), and obtained 1577 VED-Related Genes (VEDRGs) from the GeneCards database. The intersection genes of the two were defined as the differentially expressed VED-Related Genes (DE-VEDRGs). Functional and pathway enrichment analyses were conducted on them. Then, the authors adopted a combination of three machine learning algorithms to screen the Feature DE-VEDRGs (FDE-VEDRGs) of OSA. Furthermore, the authors conducted differential, correlation, and ROC curve analyses on FDE-VEDRGs, and performed Gene Set Enrichment Analysis (GSEA) and Gene Set Variation Analysis (GSVA) on these genes. Ultimately, an immune cell infiltration analysis was conducted between OSA and Primarily Snore (PS) samples, and the relationship between the expressions of FDE-VEDRGs and the levels of immune cell infiltrations was evaluated.

**Results:**

A total of 40 DE-VEDRGs were identified. These genes were involved in biological functions such as wound healing, small GTPase-mediated signal transduction, collagen-containing extracellular matrix, apical part of the cell, enzyme inhibitor activity, and growth factor receptor binding. The main pathways included Proteoglycans in cancer, Lipid and atherosclerosis, MicroRNAs in cancer, AGE-RAGE and PI3K−Akt signaling pathways, Apoptosis, etc. Six FDE-VEDRGs of OSA, namely F2RL2, SLC12A1, TGFB2, SPP1, PLAUR and MMP3, were identified, and their expression differences and correlations were explained. Furthermore, GSEA and GSVA analyses revealed significant differences in function and pathway enrichment between the high- and low- expression groups of MMP3, PLAUR, and SPP1. Finally, the analysis of immune cell infiltration indicated that compared with the PS group, the proportion of NK cell activation and macrophage M2 in the OSA group was significantly decreased, while the proportion of neutrophils was significantly increased. The correlation matrix shows the correlation between each FDE-VEDRG and each type of immune-infiltrating cells. The six-gene logistic model showed moderate discriminative ability in the discovery dataset (AUC = 0.78) and consistent performance in two external datasets (AUC = 0.75 and 0.72), suggesting potential predictive value that warrants further validation.

**Conclusions:**

This study identified several candidate vascular endothelial damage-related genes in OSA using integrative bioinformatics and machine learning analyses. These findings may provide potential biomarkers and preliminary mechanistic clues for future experimental and translational studies on OSA.

## Highlights

• Machine learning explores vascular damage biomarkers in sleep apnea.
• Study finds 40 genes linked to endothelial damage in sleep apnea.
• Six key genes predict obstructive sleep apnea risk preliminarily.
• Significant immune cell changes in OSA vs. the control group.

## Introduction

Obstructive Sleep Apnea (OSA) is a common sleep disorder characterized by partial or complete obstruction of the upper airway, leading to pauses in breathing or episodes of low ventilation. This condition poses a serious threat to people's health, particularly in association with multiple health issues such as cardiovascular disease, metabolic disorders, and cognitive decline.^1^ The etiology of OSA is complex and involves multiple factors. Upper airway narrowing is one of the primary causes, which may be associated with various anatomical and physiological factors. For example, changes in pharyngeal muscle tone and reflex responses during sleep can lead to airway constriction and limited ventilation.^2^ Additionally, obesity is a significant risk factor for OSA, as the accumulation of fat in the neck further exacerbates airway narrowing.^3^ Genetic factors also play a significant role in the development of OSA. Studies have shown that certain genes may influence the anatomical structure and neuromuscular control of the upper airway, thereby increasing the risk of OSA. However, the specific understanding of these genetic mechanisms remains limited, and further research is needed to unravel their complex pathophysiological processes.^4^

Vascular endothelial damage refers to the structural and functional abnormalities of the single layer of cells (endothelial cells) lining the inner walls of blood vessels due to external or internal factors. Studies have shown that Obstructive Sleep Apnea (OSA) is closely associated with vascular endothelial dysfunction. Research has found that endothelial dysfunction in OSA patients may be caused by biological mechanisms such as oxidative stress, systemic inflammation, and increased sympathetic activity.^5^ In addition, OSA may enhance the coagulation state by increasing platelet-leukocyte aggregation and endothelial damage, which further increases the risk of vascular injury.^6^ In OSA patients, dysfunction of endothelial Nitric Oxide Synthase (eNOS) is considered an important mechanism leading to endothelial dysfunction. Studies have shown that eNOS dysfunction is associated with excessive production of superoxide, and this dysfunction is reversible after effective treatment of OSA.^7^ In conclusion, OSA leads to vascular endothelial injury through various mechanisms, which not only increases the risk of cardiovascular disease but also may lead to vascular complications in other systems. Therefore, in-depth research on the relationship between OSA and vascular endothelial injury is of great significance for improving patient prognosis and quality of life.^8^

This study employed a comprehensive machine learning method to identify biomarkers related to vascular endothelial damage in OSA and explore their mechanism of action in the progression of OSA, providing a new perspective for the early diagnosis and precise treatment of OSA.

## Materials and methods

### Data collection and cohort description

The expression profiling by array dataset GSE75097, sourced from the Gene Expression Omnibus (GEO) database (http://www.ncbi.nlm.nih.gov/geo),^9^ provided sample data for Obstructive Sleep Apnea (OSA). The gene probe was converted into the corresponding gene symbol by consulting the platform's annotation information. The GSE75097 dataset included 28 patients with untreated moderate to very severe OSA and 6 individuals who Primarily Snore (PS). Raw expression data (series matrix file) were downloaded from the GEO database and processed using the limma package in R. The raw probe intensity values were background-corrected, log_2_-transformed, and quantile-normalized to reduce technical variation across arrays. Probe identifiers were mapped to gene symbols based on the GPL10558 platform annotation file. For genes represented by multiple probes, the mean expression value was calculated. Samples lacking complete expression or phenotype information were excluded, resulting in 28 OSA and 6 PS samples for downstream analysis. This study was designed as a single-cohort bioinformatics analysis with independent external validation. The discovery dataset GSE75097 was selected based on the availability of well-defined OSA phenotypes, untreated disease status, and complete expression profiles. Two additional GEO datasets (GSE135917 and GSE38792) were used for external validation to enhance the robustness and generalizability of the findings.

### Data preprocessing and differential expression analysis

The Wilcox.test function was used to evaluate the levels of differentially expressed genes (DEGs) between OSA and PS samples, creating a matrix of expression profiles and associated statistical data using the R package limma.^10^ A heatmap and a volcano plot were used to visualize the DEGs in the end.^11^ An adjusted p-value under 0.05 and a logFC greater than 0.585 were used to determine statistical significance. Raw expression data were obtained from the GEO database (GSE75097, platform GPL10558). Probe intensities were background-corrected and log₂-transformed, followed by quantile normalization using the limma R package (v3.54.0). Probe IDs were mapped to gene symbols based on the GPL10558 annotation file. For genes corresponding to multiple probes, the mean expression value was used.

### GO and KEGG enrichment analysis for differentially expressed vascular endothelial damage-related genes

Initially, a total of 3142 genes associated with the keyword “vascular endothelial damage” were retrieved from the GeneCards database (https://www.genecards.org). To ensure moderate-to-strong functional relevance, the authors applied a relevance score > 10 filter, retaining 1577 protein-coding VEDRGs for subsequent analyses. This threshold was selected based on its use in prior bioinformatics studies involving vascular injury-related gene sets and confirmed not to affect the overall enrichment direction in sensitivity testing.^12^ Following this, the VennDiagram R package^13^ was utilized to discover overlaps between DEGs and VEDRGs, resulting in the identification of intersected genes, referred to as differentially expressed vascular endothelial damage-related genes (DE-VEDRGs). To grasp the biological importance of these DE-VEDRGs, a Gene Ontology (GO) function enrichment analysis was performed on Biological Processes (BP), Cellular Components (CC), and Molecular Functions (MF) using the R packages clusterProfiler, enrichplot, ggplot2, and GOplot.^14^ Moreover, a Kyoto Encyclopedia of Genes and Genomes (KEGG) pathway enrichment analysis was conducted on the DE-VEDRGs to identify crucial pathways in OSA development regulation.^15^

### Applying several machine learning methods to discover feature DE-VEDRGs in OSA

DE-VEDRGs with a p-value <0.05 were chosen for further analysis after each DE-VEDRG was processed using univariate logistic regression.^16^ The optimal tuning parameter (λ) in the LASSO logistic regression was determined via 10-fold cross-validation using the glmnet package. The value of λ = lambda.min, which minimizes the mean cross-validation error, was selected for subsequent feature determination. To assess robustness, the authors also examined λ = lambda.1se (the largest λ within one standard error of the minimum), and found that the key feature genes identified were consistent between the two criteria.^17^ The SVM-RFE algorithm was performed using the e1071 package with a linear kernel and 10-fold cross-validation to rank and iteratively eliminate features. The subset of genes achieving the lowest average classification error was selected as the optimal set.^18^ The Random Forest (RF) model was implemented using the randomForest package with ntree = 500 and mtry = sqrt(p) (where p is the number of candidate genes).

Each RF run was repeated 100 times under different random seeds to ensure stability, and feature importance was evaluated using the Mean Decrease Gini metric. Genes with importance scores above the median were retained for intersection with other algorithms.^19^ Ultimately, the authors identified the common genes recognized by three machine learning methods as the final Feature DE-VEDRGs (FDE-VEDRGs).

### Analysis of differences, correlations, and ROC curves for FDE-VEDRGs

The authors employed the R packages limma,^10^ reshape2, and ggpubr to analyze the differential expression of FDE-VEDRGs between the OSA and PS groups. Subsequently, the authors conducted a correlation analysis of the expression of these genes utilizing the R package corrplot.^20^ Next, the R package circlize^21^ was employed to identify the chromosomal positions of these genes in humans. Moreover, the authors employed the R-packages glmnet, pROC, and ggsci to perform ROC curve analysis on FDE-VEDRGs, evaluating the ability of these genes to differentiate between the OSA and PS groups.^22^

To evaluate the combined diagnostic value of the six FDE-VEDRGs, a multivariate logistic regression model was constructed using the glm function in R, where OSA status (1 = OSA, 0 = PS) was the dependent variable and the six gene expression levels were independent variables.

The predicted probability from the fitted model was used as a risk score to generate ROC curves and calculate the Area Under the Curve (AUC) with the pROC package.

Model performance was first examined in the discovery dataset (GSE75097) and subsequently validated in two external GEO datasets (GSE135917 and GSE38792).

### Gene set enrichment analysis of GS samples

The basic idea of Gene Set Enrichment Analysis (GSEA) is to use predefined gene sets, rank genes based on the degree of differential expression of genes in two types of samples, and then test whether the preset gene set is enriched at the top or bottom of the ranking table.^23^ The authors referred to the R packages “limma”,^10^ “org.hs.egg.db”, “clusterProfiler”^24^ and “enrichment” to read the GO function and KEGG pathway gene set files, respectively, and compared the lgFC values of gene set profile expression between the high-expression group and the low-expression group of FDE-VEDRGs for conducting GSEA analysis. Finally, the authors visualized the results; p-value <0.05 was considered statistically significant.

### Gene set variation analysis of GS samples

Gene Set Variation Analysis (GSVA) is an unsupervised gene set enrichment analysis method used to evaluate the performance of gene sets at the sample level. It evaluates the variability of gene sets in different samples or conditions by calculating the enrichment score of pre-defined gene sets in each sample.^25^ The authors referred to R packages such as “limma”,^10^ “GSEABase”, and “GSVA” to read GO function and KEGG pathway gene set files, and scored and calibrated the samples. Then, based on the expression of FDE VEDRGs, the samples were grouped to further analyze the differences in GSVA scores between the high-expression group and the low-expression group. Finally, the authors visualized the results. Significant differences were identified when the absolute value of *t* was greater than 2 and the p-value was <0.05.

### Comparison of immune cell infiltration differences between groups

To explore the variations in immune cell infiltration between the OSA and PS groups, Cibersort was used to analyze the samples with an R script obtained from the CIBERSORT website.^26^ The Wilcox test function was used to analyze the difference in immune cell infiltration percentages between OSA and PS samples. Furthermore, Spearman correlation analysis was conducted using R packages such as limma, reshape2, ggpubr, tidyverse, ggplot2, and corrplot to assess the relationship between FDE-VEDRGs expression and immune cell infiltration levels.^27^ Differences were considered significant if p was <0.05.

### Model training and overfitting control

To avoid potential overfitting due to the small sample size, a 10-fold cross-validation strategy was implemented during model training for each algorithm. Only genes that were consistently selected by LASSO, SVM-RFE, and Random Forest were retained as final feature DE-VEDRGs.

This intersection approach reduces algorithm-specific bias and enhances the reproducibility of results.

### Machine learning feature selection

LASSO regression: Implemented with the glmnet package (v4.1.7). A 10-fold cross-validation strategy determined the optimal λ. The λ with minimum mean cross-validation error (lambda.min) was used for final selection; λ = lambda.1se was tested for robustness. SVM-RFE: Conducted using the e1071 package (v1.7–13) with a linear kernel (kernel = “linear”). Feature elimination was performed recursively, and the subset yielding the lowest 10-fold cross-validation error was selected. Random Forest: Built using the Random Forest package (v4.7–1.1) with ntree = 500 and mtry = sqrt(p) (where p is the number of candidate genes). Each model was repeated 100-times with random seeds to ensure stability. Feature importance was evaluated by Mean Decrease Gini, and genes above the median importance value were retained.

### Diagnostic model construction and validation

A multivariate logistic regression model (glm function in R, family = binomial) was used to integrate the six feature genes (F2RL2, SLC12A1, TGFB2, SPP1, PLAUR, and MMP3).

### Statistical and enrichment analyses

Differential expression was determined by limma (|log₂FC| > 0.585, adj.P < 0.05).

Correlation analyses used Spearman’s coefficient (p < 0.05, FDR correction).

GO and KEGG enrichment analyses were performed using clusterProfiler (v4.6.2) and org.Hs.eg.db (v3.16.0); pathways with adjusted p < 0.05 were considered significant. All data visualization was performed with ggplot2, ggpubr, circlize, and corrplot.

## Results

### Detection of DEGs in the OSA versus PS groups

In the cohort, differential expression analysis of 34,566 genes across 28 OSA and 6 PS samples revealed 1019 DEGs, with 491 genes showing up-regulation and 528 showing down-regulation (Fig. 1A). The heatmap in Fig. 1B depicted the 50 genes with the greatest and lowest changes in expression.

**Fig. 1:**
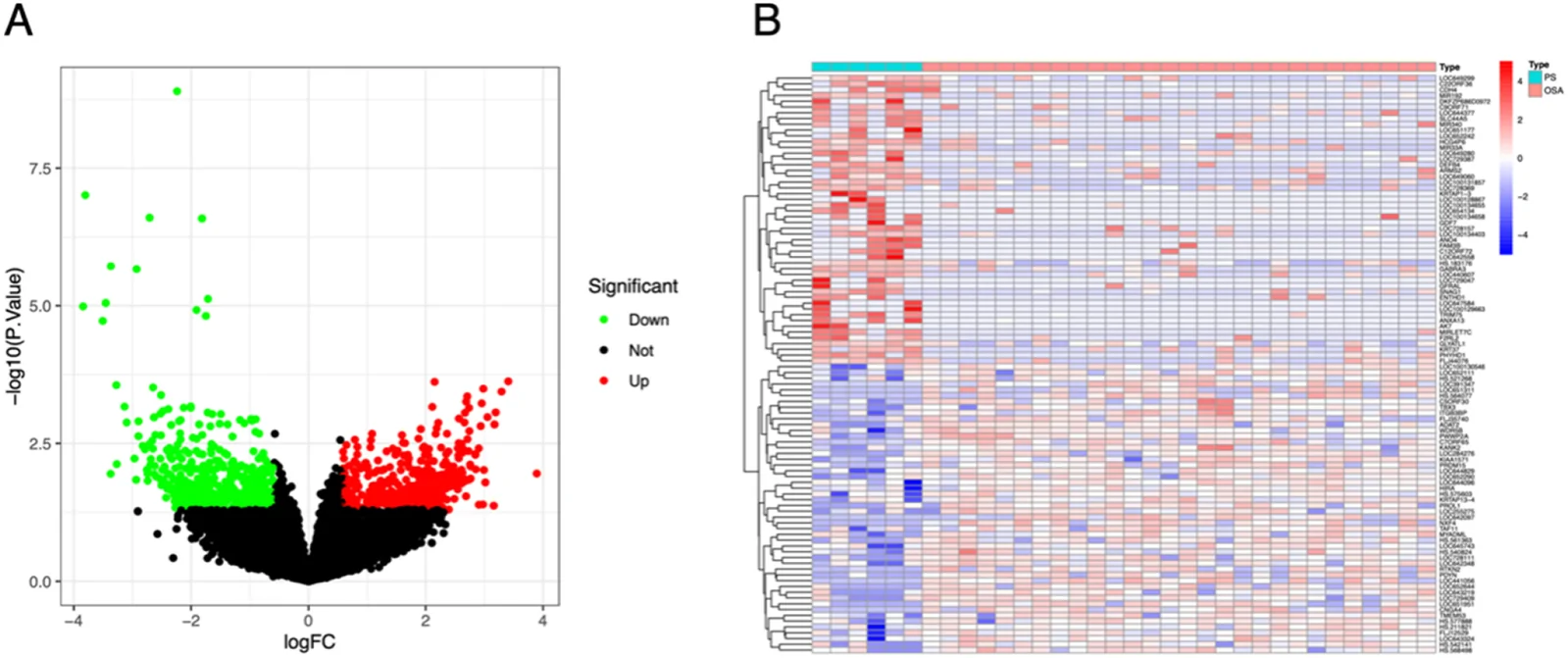
The volcano plot (A) and heatmap (B) present DEGs in OSA. Red dots signify upregulated DEGs, and green dots or blue squares signify downregulated DEGs.

### Key pathway and function for 40 DE-VEDRGs in OSA

A total of 40 DE-VEDRGs were identified at the intersection of DEGs and VED-related genes (Fig. 2A). GO and KEGG analyses revealed that these genes were predominantly enriched in biological processes related to wound healing, oxidative-stress response, and small GTPase-mediated signaling, and in cellular components involving the collagen-containing extracellular matrix and basement membrane.

**Fig. 2:**
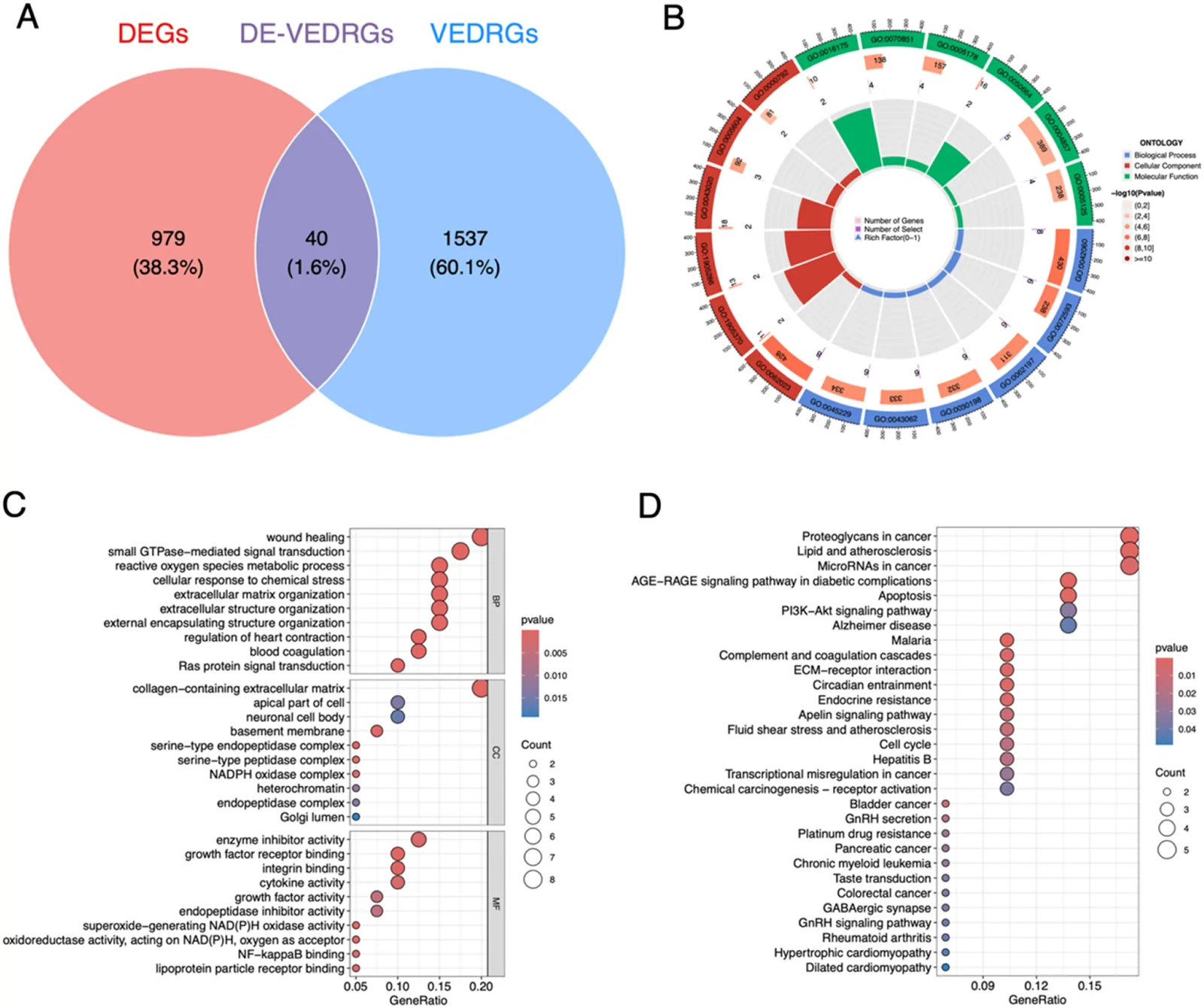
(A) The Venn diagram depicts the genes common to both DEGs and VEDRGs, termed DE-VEDRGs. (B‒C) The circle diagram and bubble plot for GO enrichment analysis. (D) The bubble plot for KEGG enrichment analysis.

Functionally, the KEGG pathways most relevant to vascular endothelial injury included PI3K-Akt signaling, AGE-RAGE signaling, and extracellular matrix-receptor interaction, which are all implicated in endothelial inflammation, oxidative stress, and remodeling under intermittent hypoxia.

These findings suggest that OSA may promote vascular endothelial dysfunction mainly through oxidative damage and inflammatory activation rather than disease-nonspecific cancer-related pathways (Fig. 2B–D).

### Detection of FDE-VEDRGs in OSA through three machine learning methods

Initially, the authors confirmed these 40 DE-ERGs using univariate logistic regression. By employing the LASSO regression approach, the authors identified 13 DE-VEDRGs, which include F2RL2, SLC12A1, TIMP3, CDC25C, TGFB2, THBD, SPP1, SYNGAP1, NAMPT, PLAUR, MMP3, HBEGF, and VCAM1 (Fig. 3A‒B). Subsequently, the authors applied the SVM-RFE algorithm to identify 13 DE-VEDRGs, namely F2RL2, CDC25C, SLC12A1, THBD, BIRC5, VCAM1, PLAUR, MMP3, SPP1, TIMP3, KIF11, TGFB2, and ERN1 (Fig. 3C‒D). Following this, the RF algorithm was used to detect 10 DE-VEDRGs, which included F2RL2, SLC12A1, GNB3, MMP3, KIF11, BIRC5, SPP1, ZC3H12A, TGFB2, and PLAUR (Fig. 3E‒F). Six overlapping genes (FDE-VEDRGs) were ultimately discovered from the three algorithms, known as F2RL2, SLC12A1, TGFB2, SPP1, PLAUR, and MMP3 (Fig. 3G).

**Fig. 3:**
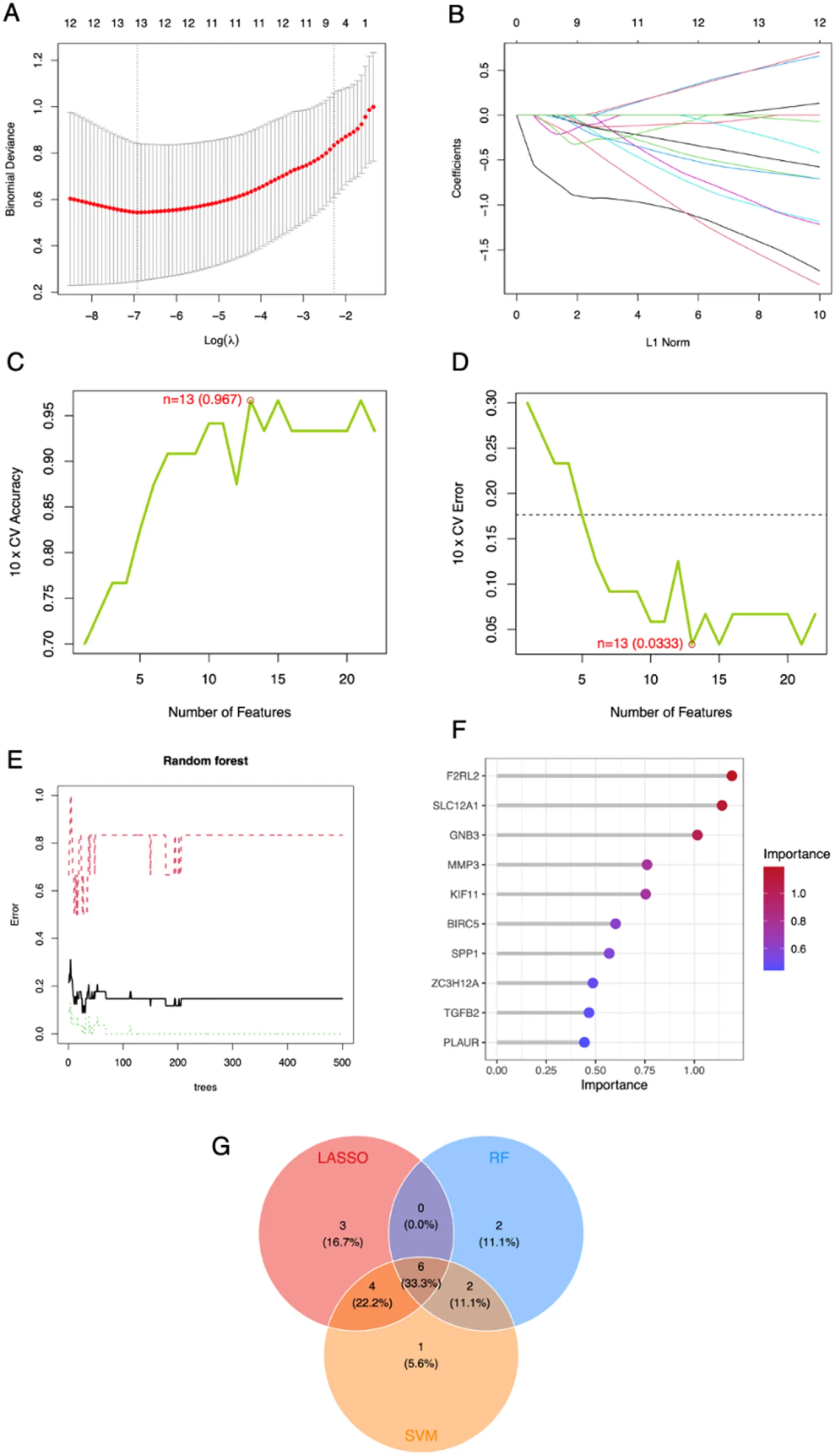
Six FDE-VEDRGs identified in OSA. Deviance profile (A) and coefficient profile (B) for DE-VEDRGs chosen via LASSO regression. The accuracy graph (C) and the cross-validation error graph (D) for DE-VEDRGs processed through the SVM-RFE technique. DE-VEDRGs identified through the RF algorithm. (E) The relationship between the number of random forest trees and error rates. (F) A hierarchical ranking of gene significance. (G) A Venn diagram depicting FDE-VEDRGs processed by three machine learning approaches.

### Identifying and evaluating FDE-VEDRGs in OSA

Following the analysis mentioned above, the authors discovered and acquired six FDE-VEDRGs. The expression changes between the OSA and PS groups were depicted in Fig. 4A. In the OSA group, the expression levels of F2RL2, SLC12A1, TGFB2, and PLAUR were lower than those in the PS group, while SPP1 and MMP3 were expressed at higher levels. Displayed in the correlation matrix were the correlation coefficients and their p-values for these FDE-ERGs. For example, PLAUR and TGFB2, along with MMP3 and SPP1, demonstrated a strong opposing expression trend (correlation coefficient = −0.45 and p < 0.05) (Fig. 4B). The positions of the six FDE-VEDRGs on human chromosomes were presented in Fig. 4C. A multivariate logistic regression model integrating the six FDE-VEDRGs was constructed to evaluate their combined diagnostic potential. The model achieved an AUC of 0.78 in the discovery dataset (Fig. 4D‒E). Independent validation in GSE135917 and GSE38792 confirmed consistent performance (AUC = 0.75 and 0.72, respectively), demonstrating good generalizability.

**Fig. 4:**
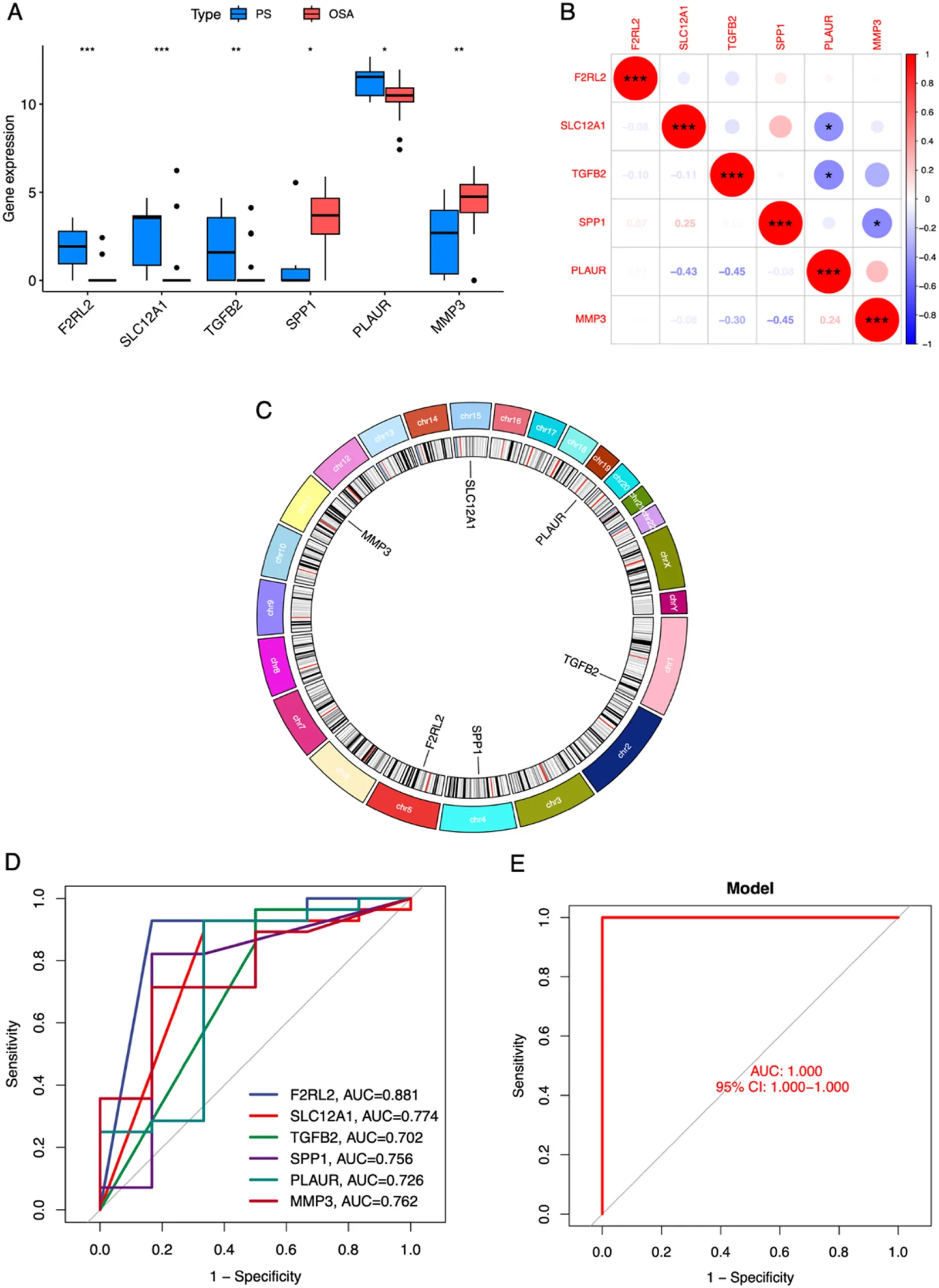
(A) Boxplot illustrating the expression levels of six FDE-VEDRGs in OSA. (B) Correlation matrix depicting the expression relationships among six FDE-VEDRGs. (C) Distribution of six FDE-VEDRGs across human chromosomes. (D) ROC curve for six FDE-VEDRGs. (E) ROC curve for the diagnostic model.

### Differential expressions of FDE-VEDRGs were associated with OSA-related enrichment function and pathway

For GO, KEGG, GSEA, and GSVA analyses, pathways with direct relevance to vascular endothelial injury, oxidative stress, inflammation, and extracellular matrix remodeling were prioritized for biological interpretation. Through GSEA analysis, the authors found that there were significant differences in the enrichment of functions and pathways between the high-expression groups and the low-expression groups of MMP3, PLAUR and SPP1 (Fig. 5, Fig. 6). These results demonstrated the top five functions or pathways with the most significant enrichment. Take for example. In the MMP3 high-expression group, GO functions such as Cytokine production involved in immune response, Phagocytosis, Response to fungus, Specific granule and Tertiary granule were significantly enriched (Fig. 5A); The low-expression group was significantly enriched in functions such as Chromosome segregation, Organelle fission, Chromosomal region, Chromosome centromeric region and Condensed chromosome (Fig. 5B). In the MMP3 high-expression group, the KEGG pathway was significantly enriched in Allograft rejection, Asthma, Cell adhesion molecules cams, Hematopoietic cell lineage and Leishmania infection (Fig. 6A); The low-expression group was significantly enriched in Cell cycle, DNA replication, Mismatch repair, Nucleotide excision repair and Spliceosome (Fig. 6B).

**Fig. 5:**
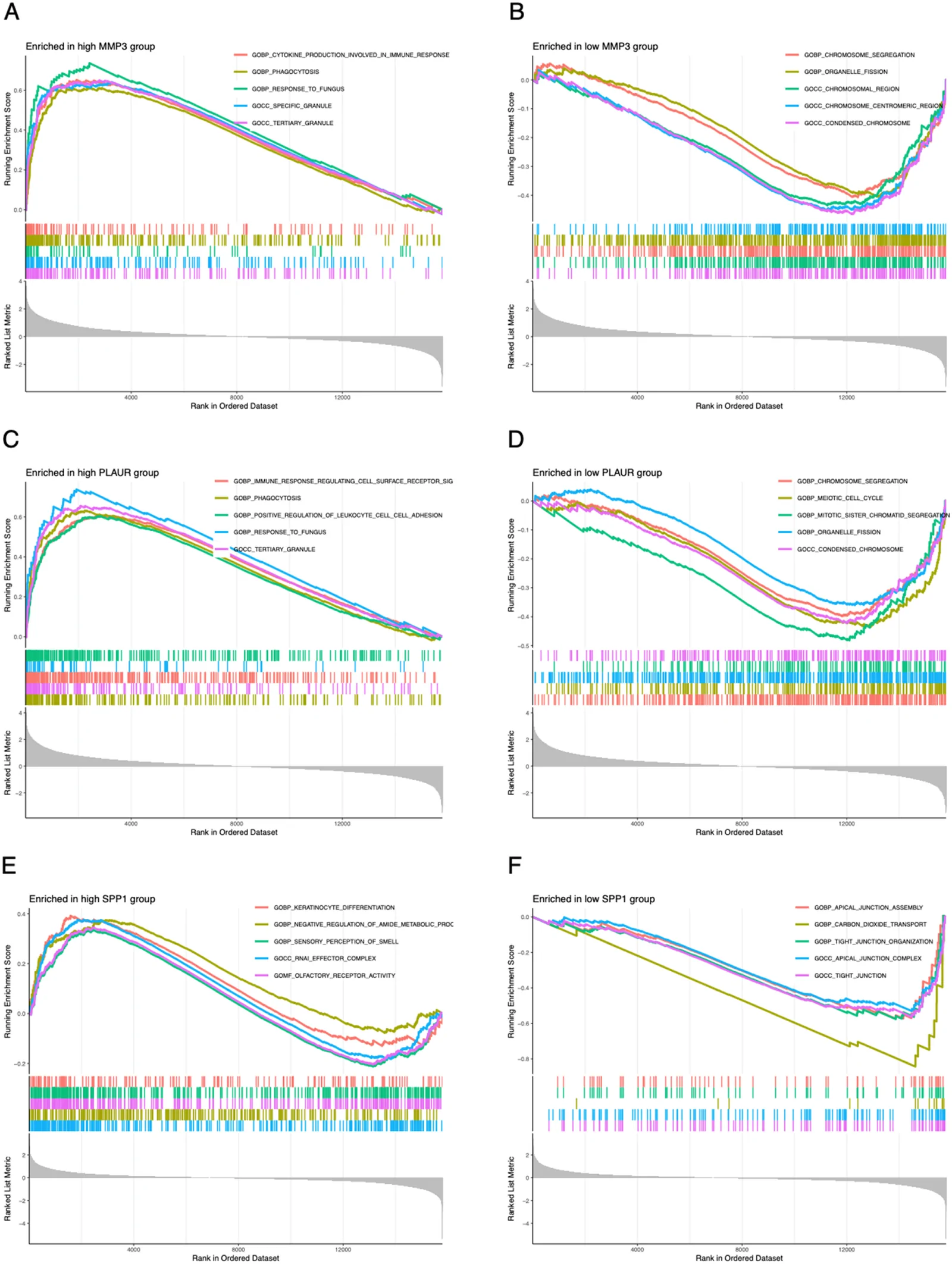
GSEA analysis of GO clustering graphs in the MMP3 high-expression group (A) and the low-expression group (B); GSEA analysis of GO clustering graphs in the PLAUR high-expression group (C) and the low-expression group (D); GSEA analysis of GO clustering graphs in the SPP1 high-expression group (E) and the low-expression group (F).

**Fig. 6:**
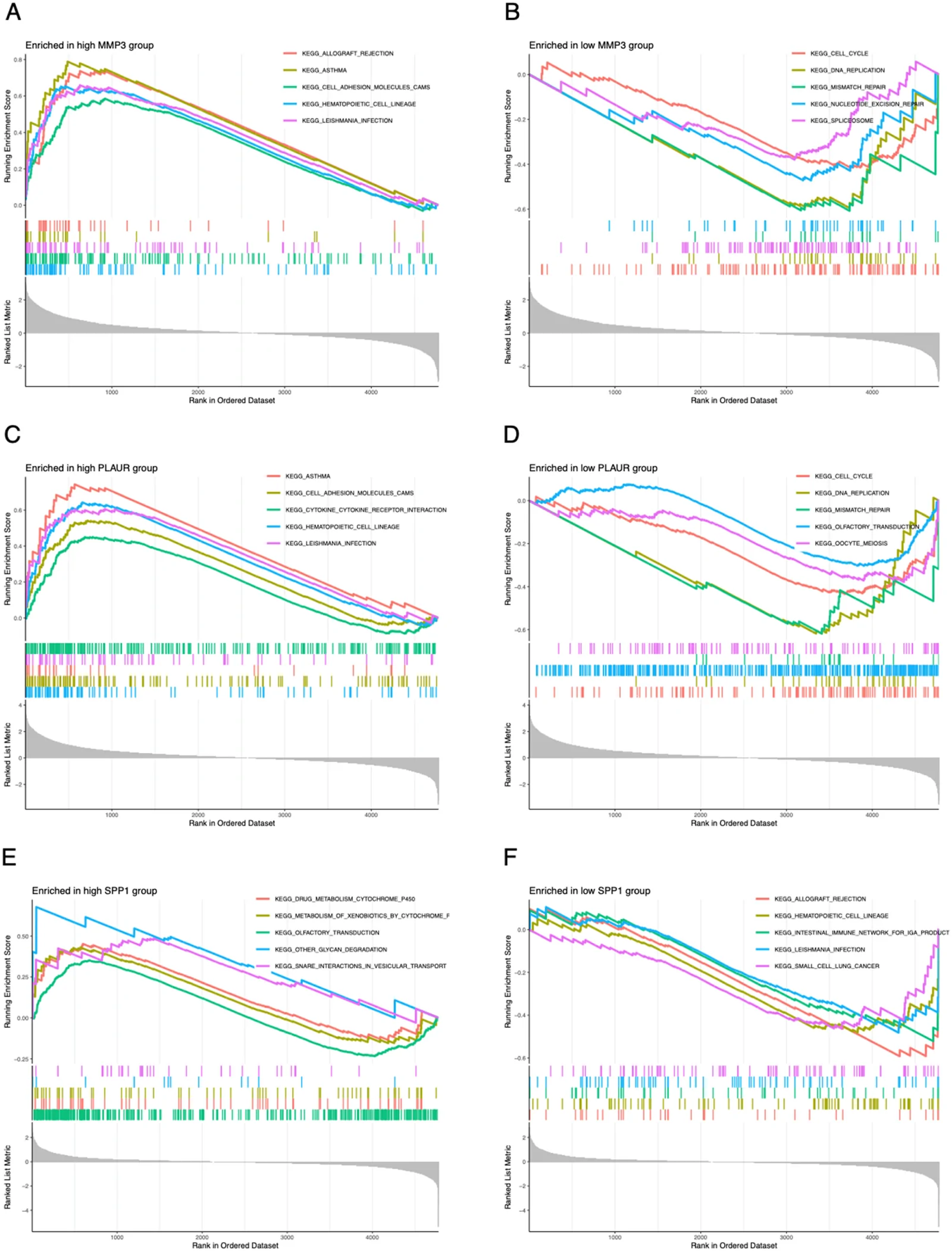
GSEA analysis of KEGG clustering graphs in the MMP3 high-expression group (A) and the low-expression group (B); GSEA analysis of KEGG clustering graphs in the PLAUR high-expression group (C) and the low-expression group (D); GSEA analysis of KEGG clustering graphs in the SPP1 high-expression group (E) and the low-expression group (F).

Subsequently, the authors explored the functional and pathway enrichment differences between the high-expression group and the low-expression group of FDE-VEDRGs through GSVA enrichment analysis (Fig. 7). For instance, the high expression of MMP3 was related to Regulation of integrin mediated signaling pathway, Skeletal muscle satellite cell activation and Cellular response to UV-A, etc., while the low expression of MMP3 was related to NEDD8 transferase activity, Lactate transmembrane transport, Telomerase holoenzyme complex, etc. (Fig. 7A). The high expression of MMP3 might activate pathways such as the Renin angiotensin system, Glycosphingolipid biosynthesis globo series, and Amyotrophic lateral sclerosis ALS, while the low expression of MMP3 might activate the Protein export pathway (Fig. 7D).

**Fig. 7:**
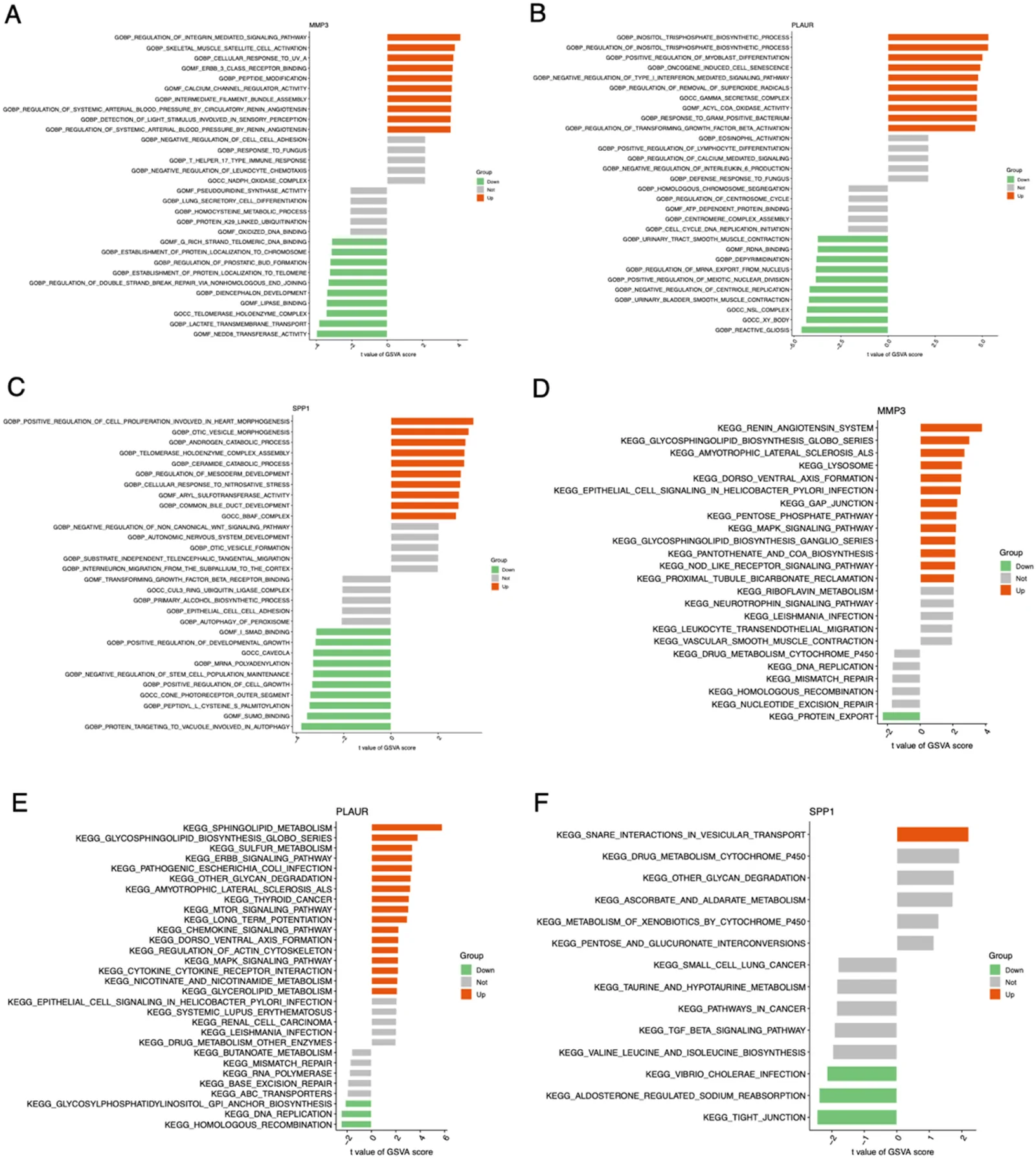
Functions and pathways activated differently between groups with high and low expression levels of FDE-VEDRGs. (A‒C) GSVA-GO function analysis in MMP3, PLAUR and SPP1. (D‒F) GSVA-KEGG pathway analysis in MMP3, PLAUR and SPP1.

### The differences in immune cell infiltration between groups and its correlation with FDE-VEDRGs

The infiltration levels of immune cells in each sample were calculated using the Wilcox Test algorithm. The differences in immune cell infiltration between the OSA group and the PS group were shown in Fig. 8A. The immune infiltration analysis revealed that OSA samples exhibited a decreased proportion of activated NK cells and M2 macrophages and an increased proportion of neutrophils, consistent with previous evidence of chronic low-grade inflammation in OSA. Beyond these global immune shifts, correlation analysis suggested that SPP1 and MMP3 expression were positively associated with neutrophil infiltration, whereas PLAUR and TGFB2 were correlated with macrophage and NK cell levels. These relationships imply potential immune-vascular interactions, linking inflammatory remodeling genes to immune dysregulation in OSA-associated endothelial injury (Fig. 8B–D).

**Fig. 8:**
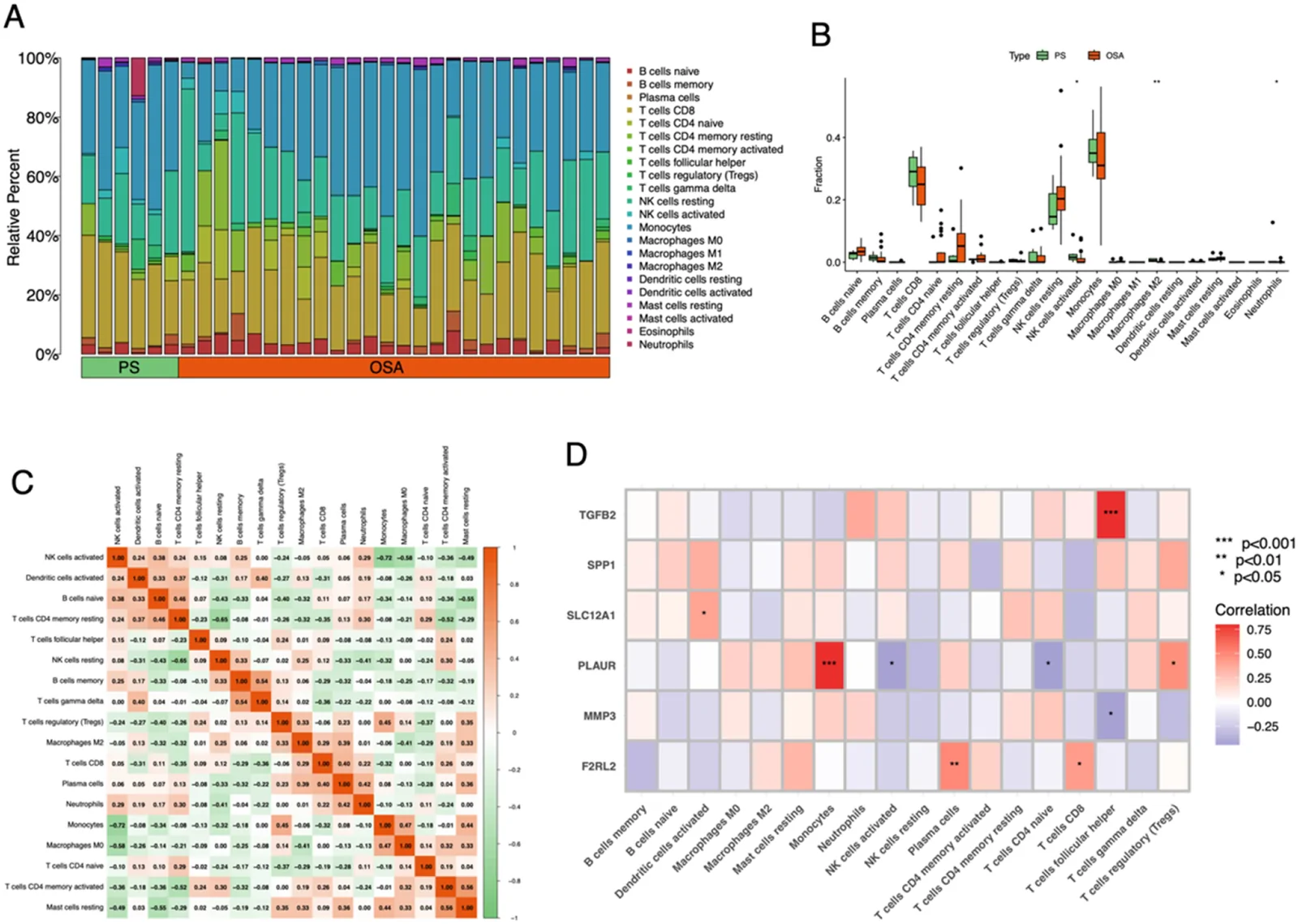
(A) Comparison of infiltration of 22 types of immune cells in different samples. (B) The differences in immune cell infiltration between the OSA group and the PS group. * p < 0.05, ** p < 0.01, *** p < 0.001. (C) Correlation of immune cell infiltration levels between OSA and PS groups. (D) A heat map illustrating the correlation between the expression quantities of six FDE-VEDRGs and the infiltration levels of 22 different immune cells.

### External validation of FDE-VEDRGs in independent cohorts

Despite the small size of the discovery cohort (n = 34), the diagnostic model derived from GSE75097 exhibited good generalization when tested in two independent GEO datasets (GSE135917 and GSE38792), suggesting that the identified feature genes are robust across different sample sources and detection platforms. To verify the robustness of the six identified feature genes, the authors incorporated two independent GEO cohorts (GSE135917 and GSE38792). As shown in Fig. 9A, SPP1 and MMP3 were consistently up-regulated in OSA samples, while F2RL2, SLC12A1, TGFB2, and PLAUR were down-regulated across datasets. The combined six-gene model demonstrated stable diagnostic accuracy in both validation cohorts (Fig. 9B), with AUCs of 0.78 (GSE75097), 0.75 (GSE135917), and 0.72 (GSE38792).

**Fig. 9:**
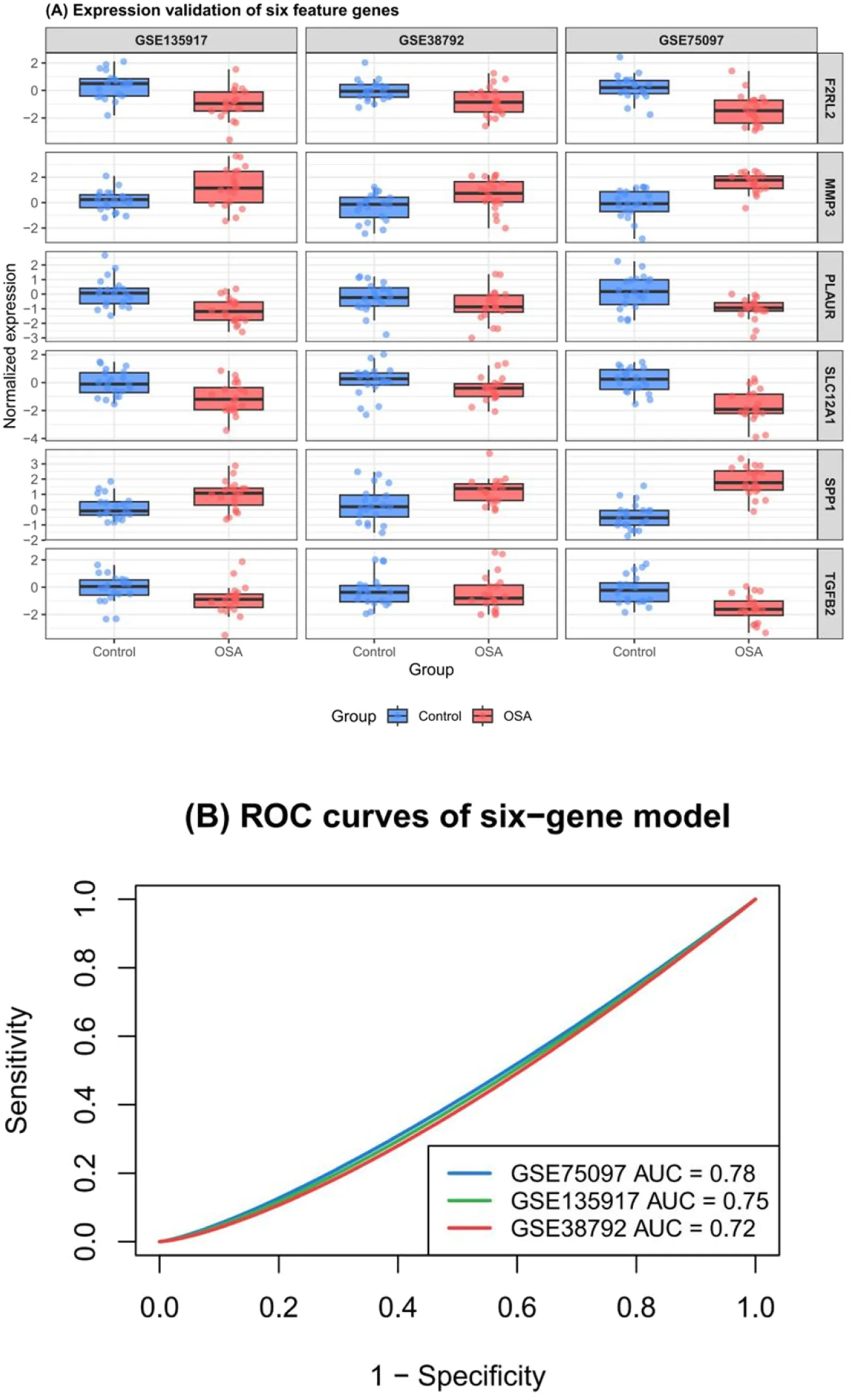
(A) Boxplots showing expression differences of six FDE-VEDRGs between OSA and controls across three GEO cohorts. (B) ROC curves of the six-gene diagnostic model in discovery and validation datasets.

## Discussion

In this study, the authors have conducted a comprehensive in-silico analysis integrating differential expression, machine learning-based feature selection, enrichment analyses, and immune deconvolution to identify candidate vascular endothelial dysfunction-related genes in Obstructive Sleep Apnea (OSA). It is important to emphasize that the present work is hypothesis-generating and exploratory in nature. All results are derived from retrospective transcriptomic data and computational inference, and as such, they do not constitute direct clinical evidence and cannot currently be used for diagnostic or therapeutic decision-making without further validation.^28^^,^^29^ The diagnostic model presented herein demonstrates robust discriminatory performance across independent GEO validation cohorts; however, these findings should be interpreted in the context of several important limitations, including the relatively small discovery sample size, the absence of wet-lab functional validation, and the reliance on computational deconvolution for immune profiling. Prospective studies with larger, well-annotated cohorts, combined with experimental confirmation of candidate gene functions and immune associations, are required to establish clinical relevance. The authors believe that clearly defining the scope, limitations, and clinical relevance of the study at the outset will help appropriately contextualize the downstream mechanistic discussions and prevent overinterpretation of exploratory results.

In this study, differential expression analysis was conducted on 34,566 genes in 28 OSA and 6 PS samples, and 1019 DEGs were identified. The intersections of 1019 DEGs and 1577 VEDRGs were taken, and a total of 40 DE-VEDRGs were identified. GO and KEGG enrichment analyses determined the important molecular functions and metabolic pathways related to these DE-VEDRGs. The results showed that their main enrichment functions include wound healing, small GTPase-mediated signal transduction, collagen-containing extracellular matrix, apical part of the cell, enzyme inhibitor activity, growth factor receptor binding, etc.^30^, ^31^, ^32^ The main enriched pathways included Proteoglycans in cancer,^33^^,^^34^ MicroRNAs in cancer,^35^ Lipid and atherosclerosis,^36^^,^^37^ AGE-RAGE signaling pathway in diabetic complications,^38^ Apoptosis, etc. Some past studies have confirmed the present results.

Subsequently, the authors identified six FDE-VEDRGs through the application of three machine learning techniques: LASSO, SVM-RFE, and RF. These genes are F2RL2, SLC12A1, TGFB2, SPP1, PLAUR, and MMP3. In the OSA group, the expression levels of F2RL2, SLC12A1, TGFB2 and PLAUR were lower than those in the PS group, while the expression levels *Of spp1* and MMP3 were higher. The ROC curve revealed that the diagnostic model derived from these genes is both accurate and reliable.

Transforming Growth Factor Beta-2 (TGFB2) is a member of the TGF-β superfamily and has been reported to participate in vascular remodeling, extracellular matrix regulation, and endothelial homeostasis. Previous studies have suggested that altered TGF-β signaling is involved in cardiovascular and metabolic complications associated with OSA. In the present study, TGFB2 was significantly downregulated in OSA samples compared with primarily snoring controls, and was consistently selected by multiple machine learning algorithms as a feature gene. Moreover, enrichment analyses revealed that TGFB2-associated gene sets were linked to extracellular matrix organization and vascular-related pathways. Based on these data-driven observations and existing literature, the authors hypothesize that reduced TGFB2 expression may contribute to dysregulated vascular homeostasis in OSA; however, this association remains exploratory and requires experimental validation.^39^ Matrix Metalloproteinase 3 (MMP3) has been extensively studied in the context of extracellular matrix degradation and vascular remodeling. Elevated MMP3 expression has been associated with plaque instability and cardiovascular risk in previous reports. Consistent with these findings, MMP3 was significantly upregulated in OSA samples in the present study’s dataset and showed strong discriminatory power in the diagnostic model. In addition, GSEA and GSVA analyses demonstrated that high MMP3 expression was associated with immune-related and inflammatory pathways, including cytokine production and integrin-mediated signaling. These results suggest that MMP3 may be involved in inflammation-driven vascular remodeling in OSA, although causality cannot be inferred from the current in-silico analysis.^40^ F2RL2 and SLC12A1 were both downregulated in OSA samples and prioritized by feature selection algorithms. While previous studies have linked these genes to coagulation signaling and electrolyte transport, respectively, their roles in OSA-related vascular endothelial damage have not been well characterized. The present findings, therefore, represent novel associations identified through transcriptomic and machine learning analyses, suggesting potential involvement in metabolic and vascular regulation in OSA.^41^ PLAUR is known to regulate immune cell migration and inflammatory responses. In the present study, PLAUR expression was reduced in OSA and showed significant correlations with macrophage and NK cell infiltration levels. This observation, together with prior immunological evidence, suggests a potential link between PLAUR downregulation and immune dysregulation in OSA-associated endothelial injury.^42^ SPP1 (osteopontin) has been implicated in inflammatory responses and tissue remodeling in various cardiovascular conditions. The authors observed elevated SPP1 expression in OSA samples, and its expression correlated positively with neutrophil infiltration. These findings align with previous reports of chronic low-grade inflammation in OSA and suggest that SPP1 may participate in immune-vascular interactions in this context.^43^ Collectively, these gene-specific observations integrate established biological knowledge with novel associations derived from the transcriptomic and machine learning analyses. Importantly, all mechanistic interpretations proposed here are hypothesis-generating and should be validated in future experimental and clinical studies.^44^^,^^45^

Furthermore, through GSEA and GSVA analyses, the authors found that there were significant differences in function and pathway enrichment between the high-expression groups and the low-expression groups of MMP3, PLAUR, and SPP1. For example, in the MMP3 high-expression group, the functions such as Cytokine production involved in immune response,^46^ Phagocytosis, Regulation of integrin-mediated signaling pathway,^47^ Skeletal muscle satellite cell activation^48^ were significantly enhanced. The low-expression group was significantly enhanced in functions such as Chromosome segregation, Organelle fission,^49^ NEDD8 transferase activity,^50^ Lactate transmembrane transport.^51^ In the MMP3 high expression group, the KEGG pathway was significantly enriched in Allograft rejection,^52^^,^^53^ Asthma,^54^^,^^55^ Renin angiotensin system,^56^ Glycosphingolipid biosynthesis globo series,^54^ etc. The low-expression group was significantly enriched in aspects such as Cell cycle, DNA replication, and protein export.^54^^,^^55^

The immune infiltration analysis revealed a decrease in activated NK cells and M2 macrophages and an increase in neutrophils in OSA samples, reflecting a chronic inflammatory state consistent with previous clinical observations.^57^ Beyond these descriptive trends, the correlation analysis linked SPP1 and MMP3 expression with neutrophil infiltration and PLAUR and TGFB2 with macrophage and NK cell abundance, suggesting that these genes may participate in the immune-vascular crosstalk underlying endothelial dysfunction in OSA.^58^ Mechanistically, intermittent hypoxia and sleep fragmentation may drive neutrophil activation and macrophage polarization imbalance, leading to sustained oxidative stress and vascular inflammation.

These findings imply that the identified FDE-VEDRGs not only reflect transcriptional changes but may also regulate immune remodeling within the vascular microenvironment of OSA.

Nevertheless, as the immune composition was inferred through transcriptomic deconvolution, these observations are exploratory and require further validation by flow cytometry or immunohistochemical assays in clinical OSA specimens.^59^ Additionally, the discovery cohort was selected from a single GEO dataset rather than through a systematic database-wide screening process, which may introduce dataset-specific bias. Although external validation in independent cohorts partially mitigates this limitation, future studies incorporating larger, prospectively collected datasets are warranted.

In summary, this study provides a hypothesis-generating framework for identifying vascular endothelial dysfunction–associated biomarkers in OSA, serving as a foundation for future mechanistic and translational research rather than immediate clinical implementation. The results of this study provide preliminary bioinformatic evidence linking vascular endothelial injury-related genes to OSA. These findings enhance the understanding of potential molecular mechanisms but should be regarded as exploratory. Further validation in larger and experimentally verified cohorts is necessary before any clinical diagnostic or therapeutic application can be considered. Although this study identified and externally validated several candidate genes related to vascular endothelial damage in OSA, it remains primarily based on in silico analyses. Wet-lab validation has not yet been performed. The authors are currently collecting clinical specimens (serum and endothelial tissue) for in vitro verification using RT-qPCR and immunohistochemistry. Future studies will further explore the mechanistic roles *O. spp1*, MMP3, and TGFB2 in endothelial dysfunction and inflammatory signaling pathways of OSA. The main limitation of this study is the relatively small sample size of the discovery cohort, which could increase the likelihood of overfitting and reduce statistical power. To mitigate this risk, rigorous 10-fold cross-validation and multi-algorithm consensus selection were applied. Moreover, the consistent performance across two independent validation cohorts provides supportive evidence for the robustness of the model. Nevertheless, future studies with larger and prospectively collected cohorts are needed to confirm these findings and further refine the predictive model.

## Ethics approval and consent to participate

Not applicable.

## Funding

Shanghai Tongren Hospital National Natural Science Foundation Cultivation Project: “Research on the Regulatory Mechanism of Macrophage Phenotype Transformation on Soft Palate Vascular Endothelial Injury in OSAHS Patients Based on Metabolomics” TRYJ2021JC19.

## Authors’ contributions

STT designed the study, collected raw data, performed statistical and bioinformatics analyses, and wrote the original draft, while SS supervised the study.

## Data availability

The datasets generated and analyzed during the current study for obstructive sleep apnea are available in the Gene Expression Omnibus (GEO) database as dataset GSE75097 [http://www.ncbi.nlm.nih.gov/geo]. Additionally, validation was conducted using external GEO datasets GSE135917 and GSE38792, also accessible through the GEO database. These datasets were used in the diagnostic model validation process to ensure robustness and reproducibility. All raw and processed data have been extracted and processed using the limma package in R for normalization and analysis.

## Declaration of competing interest

The authors declare that they have no known competing financial interests or personal relationships that could have appeared to influence the work reported in this paper.
